# Validation of depression determinants in caregivers of dementia patients with machine learning algorithms and statistical model

**DOI:** 10.3389/fmed.2023.1095385

**Published:** 2023-02-02

**Authors:** Kangrim Cho, Junggu Choi, Sanghoon Han

**Affiliations:** ^1^Yonsei Graduate Program in Cognitive Science, Yonsei University, Seoul, Republic of Korea; ^2^Department of Psychology, Yonsei University, Seoul, Republic of Korea

**Keywords:** depression, dementia, caregiver, community health survey, machine learning, statistical model

## Abstract

**Introduction:**

Due to its increasing prevalence, dementia is currently one of the most extensively studied health issues. Although it represents a comparatively less-addressed issue, the caregiving burden for dementia patients is likewise receiving attention.

**Methods:**

To identify determinants of depression in dementia caregivers, using Community Health Survey (CHS) data collected by the Korea Disease Control and Prevention Agency (KDCA). By setting “dementia caregiver's status of residence with patient” as a standard variable, we selected corresponding CHS data from 2011 to 2019. After refining the data, we split dementia caregiver and general population groups among the dataset (*n* = 15,708; common variables = 34). We then applied three machine learning algorithms: Extreme Gradient Boosting (XGBoost), Logistic Regression (LR), and Support Vector Classifier (SVC). Subsequently, we selected XGBoost, as it exhibited superior performance to the other algorithms. On the feature importance of XGBoost, we performed a multivariate hierarchical regression analysis to validate the depression causes experienced in each group. We validated the results of the statistical model analysis by performing Welch's *t*-test on the main determinants exhibited within each group.

**Results:**

By verifying the results from machine learning *via* statistical model analysis, we found “sex” to highly impact depression in dementia caregivers, whereas “status of economic activities” is significantly associated with depression in the general population.

**Discussion:**

The evident difference in causes of depression between the two groups may serve as a basis for policy development to improve the mental health of dementia caregivers.

## 1. Introduction

Owing to its increasing prevalence, dementia is considered one of the most crucial health issues worldwide. Nichols et al. found that the global prevalence of dementia increased by 117% from 1990 to 2016 across 195 countries (1), and they predicted a threefold increase in the number of dementia patients from 2019 to 2050 (2). The socioeconomic costs of dementia have likewise increased. A global study on the economic burden of dementia discovered a total increase from $279.6 billion in 2000 to $946 billion in 2016, with a rapid annual increase rate of 15.94% (3). Furthermore, this economic burden poses a more significant issue in low- and middle-income countries (LMICs). Mattap et al. analyzed the total national costs of dementia in 122 LMICs, and found them to range from 1.04 to $195 million, accounting for 0.45% of each country's GDP on average (4). The impact of dementia has been emphasized by numerous studies analyzing its increasing prevalence and burden. However, research pertaining to dementia caregivers is relatively less prominent.

The burden on dementia caregivers can be investigated in terms of various aspects. Due to the heavy strain of caring for dementia patients, caregivers often suffer a severe psychological burden. In one related study, Givens et al. found a stronger prevalence of high depressive symptoms in caregivers of dementia patients compared to those of patients with other illnesses (5). In addition to its psychological effects, the caregiver burden also has a significant socioeconomic impact. Financial strain, which entails one's “perceived economic stress and lack of economic support” (6), is found to be positively associated with the load of the caregiving role in dementia caregivers (7). The aforementioned studies illustrate the severe impact of caregiver burden in psychological as well as socioeconomic contexts.

The psychological burden of caregivers has generally been analyzed by statistical methods. An ordinary least squares regression was employed to examine the family caregiver burdens among the Chinese population (8), whereas multivariate linear and logistic regressions were applied to determine the variables affecting the domestic dementia caregiver burden in South Korea (9). Recently, machine learning has been employed as an analytical tool to identify relations between diverse variables. For example, Antoniadi et al. employed random forests to predict the psychological burden of amyotrophic lateral sclerosis (ALS) caregivers (10). Subsequently, they employed Extreme Gradient Boosting (XGBoost), an explainable machine learning algorithm, to identify the predictors of the ALS caregivers' quality of life (QoL) indices (11).

Based on the aforementioned studies, we identified and analyzed the factors associated with depression in dementia caregivers using machine learning algorithms. We examined various determinants and features using a large-scale dataset collected nationwide in South Korea. To ensure more accurate analysis, we validated the results obtained from machine learning with a statistical model.

## 2. Methods

### 2.1. Overview

**Figure 1:**
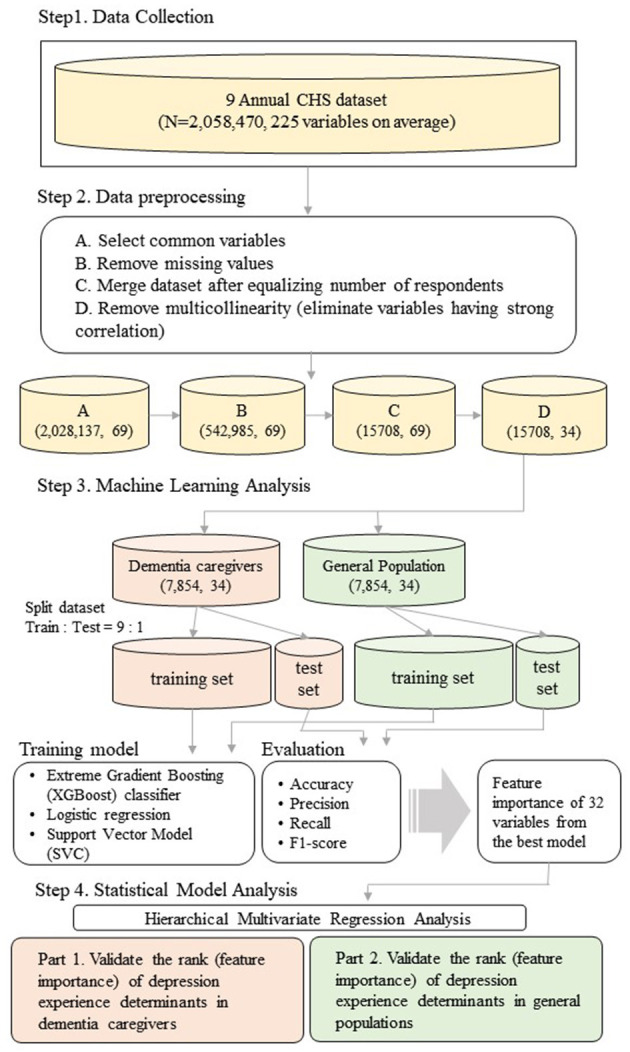
Overview of the research analysis procedure.

To identify the determinants of depression in dementia caregivers, we compared them against those appearing within the general population. First, we conducted an analysis using data from the Community Health Survey (CHS), collected by the Korea Disease Control and Prevention Agency (KDCA) (12). Because “dementia caregiver's status of residence with the patient” is the standard variable of this study, we selected an appropriate dataset ranging from 2011 to 2019 (Step 1). Subsequently, we preprocessed the collected dataset, wherein any inappropriate data were removed prior to analysis (Step 2). Based on the variables represented in the survey, we applied XGBoost, Logistic Regression (LR), and Support Vector Classifier (SVC) to determine factors associated with depression in dementia caregivers (Step 3). Finally, we validated the results from machine learning by conducting a hierarchical multivariate regression analysis (Step 4). Details of the procedure are illustrated in Figure 1.

### 2.2. Dataset

The dataset analyzed in this study comprises open-access data from the CHS, a nationwide annual survey conducted by the KDCA to construct a fundamental basis of health policy and compare the health statuses between regions in South Korea (12). This survey examines various health issues affecting South Korean citizens over the age of 19. The questions within the survey pertain to physical as well as mental health, encompassing ~ 225 variables on average. The number of respondents remained similar among all surveys, with an average of ~ 228,719. A detailed description of the dataset is presented in Table 1.

**Table 1:** Baseline characteristics of the CHS dataset.

**Characteristics**		**CHS**
Number of respondents (*N*)		2,058,470
Age, Mean (SD)		52.8 (17.32)
Gender	Male (%)	925,348 (44.95)
	Female (%)	1,133,122 (55.05)
Status of economic activities	Yes (%)	1,291,070 (62.72)
	No (%)	766,719 (37.25)
	N/A (%)	681 (0.03)
Subjective health status	Extremely good (%)	115,396 (5.61)
	Good (%)	646,090 (31.39)
	Normal (%)	859,675 (41.76)
	Bad (%)	340,713 (16.55)
	Extremely bad (%)	96,379 (4.68)
	N/A (%)	217 (0.01)
Doctor's diagnosis on hypertension	Yes (%)	528,329 (25.67)
	No (%)	1,529,694 (74.31)
	N/A (%)	447 (0.02)
Doctor's diagnosis on Diabetes	Yes (%)	206,104 (10.01)
	No (%)	1,851,828 (89.96)
	N/A (%)	538 (0.03)
Dementia caregiver's status of residence with the patient	Yes (%)	24,287 (1.18)
	No (%)	1,602,265 (77.85)
	N/A (%)	431,490 (20.97)

### 2.3. Preprocessing

Prior to analysis, we preprocessed the dataset in four steps. First, any uncommon variables were eliminated from the set. To ensure that all data reflected the standard variable, the original range from 2008 to 2020 was narrowed down to a span from 2011 to 2019. Consequently, the dataset's dimensionality was reduced to (2,028,137, 69). Certain variables were associated with different variable codes or data types and were appropriately equalized. Next, we eliminated inappropriate responses [i.e., no response, not applicable(N/A)], and this step decreased the total number of respondents from 2,058,470 to 542,985, which is 26.37% of total original respondents. After equalizing the number of respondents on the “dementia caregiver's status of residence with the patient” question, all nine annual datasets were merged. A total of 7,854 respondents answered “yes” to this question, whereas 417,272 participants answered “no.” Due to the significant gap between the two responses, we applied equalization to prevent potential bias prior to analysis. By randomly selecting the larger group, the dataset's dimensionality was reduced to (15,708, 69). Lastly, we eliminated the variables that exhibited strong correlations between themselves, thereby preventing distortion due to multicollinearity. To achieve this, we composed a correlation matrix of variables and eliminated the former variable with a threshold value of 0.4. Consequently, the dimensionality of the final dataset shrunk to (15,708, 34). Variables present in the final dataset are listed in Table 2.

**Table 2:** List of common variables within the final dataset.

**No**.	**Variable**	**Variable name**	**Type^a^**	**No**.	**Variable**	**Variable name**	**Type**
1	town_t	dong/eup, myeon^b^	Categorical (1)	18	orb_01z1	Experience of chewing discomfort	Categorical (3)
2	apt_t	Type of Residence	Categorical (1)	19	ore_02z1	Experience of required dental care receipt failure	Categorical (2)
3	Sex	Sex	Categorical (1)	20	mta_01z1	Subjective stress status	Categorical (3)
4	Age	Age	Continuous	21	mtb_01z1	Depression Experiences	Categorical (2)
5	fma_24z1	Household Income	Categorical (4)	22	mtc_01z1	Average sleeping time	Continuous
6	fma_01z1	Number of households	Continuous	23	sca_01z1	Status of influenza vaccination	Categorical (2)
7	fma_04z1	Status of beneficiary of national basic livelihood	Categorical (2)	24	hya_04z1	Doctor's diagnosis on hypertension	Categorical (2)
8	fma_18z1	Dementia caregiver's status of residence with the patient	Categorical (2)	25	dia_04z1	Doctor's diagnosis on diabetes	Categorical (2)
9	qoa_01z1	Subjective health status	Categorical (3)	26	sra_01z1	Experience of required medical service receipt failure	Categorical (2)
10	dra_01z1	Lifelong status of alcohol drinking	Categorical (2)	27	ira_01z1	Experiences of accident/addiction a year	Categorical (2)
11	sfa_01z1	Status of car driving	Categorical (2)	28	ira_02z1	Frequency of accident/addiction a year	Continuous
12	sfb_07z1	Experience of riding in a car driven by a drunk person	Categorical (2)	29	qoc_05z1	EQ-5D anxiety/depression	Categorical (3)
13	sfa_05z1	Status of motorcycle driving	Categorical (2)	30	soe_01z1	Residence period_city/province	Categorical (4)
14	phb_01z1	Days of walking per week	Continuous	31	soa_01z1	Status of economic activities	Categorical (2)
15	oba_01z1	Subjective body frame	Categorical (3)	32	sob_01z1	Most recent educational background	Categorical (1)
16	obb_01z1	Experience in weight management	Categorical (1)	33	sob_02z1	Graduation status	Categorical (1)
17	ora_01z1	Subjective oral health status	Categorical (3)	34	sod_02z2	Marriage status	Categorical (1)

^a^Categorical (1): option decision, distinctive options on each question were given (e.g., “urban” or “rural”);

Categorical (2): Yes/No;

Categorical (3): Index/Scale, options on scales were given (e.g., “hardly ever,” “rarely,” “often,” “usually,” “always”);

Categorical (4): Range, options on range for each question were provided (e.g., “under 500,000 KRW” or “500,000 ~ 1,000,000 KRW”).

^b^A regional system unit in Korea that indicates towns.

### 2.4. Machine learning algorithms

We trained three machine learning models using the preprocessed dataset: XGBoost, LR, and SVC. The XGBoost algorithm is an ensemble model of decision trees. Rather than employing a single strong decision tree, this algorithm weighs several weak decision trees sequentially, thereby constructing a strong integrated model (13). With each newly added classifier, the algorithm works to minimize the loss:


(1) $$\begin{array}{l} L\left(\phi \right)=\underset{i}{\sum }l\left(\;\hat{y_{i}}\;,\;y_{i}\right)+\underset{k}{\sum }\Omega \left(f_{k}\right) \end{array}$$

(2) $$\begin{array}{l} where\Omega \left(f\right)=\gamma T+\frac{1}{2}\lambda ||\omega |{|}^{2} \end{array}$$

(3) $$\begin{array}{l} \hat{y_{i}}=\phi \left(x_{i}\right)=\overset{K}{\underset{k=1}{\sum }}f_{k}\left(x_{i}\right),f_{k}\in F \end{array}$$

The algorithm trains decision trees with n samples and m features in dimension $D=\left\{\left(x_{i},y_{i}\right)\right\}\left(|D|=n,x_{i}\in R^{m},y_{i}\in R\right)$. In this study, we used two sets comprising 7,854 (n) samples and 32 (m) features to train the model. Formula (1) expresses the loss function of the XGBoost algorithm. To optimize the loss, we minimized the gap between the predicted ($\hat{y_{i}}\;$) and real (*y*_*i*_) values. Formula (2) represents a penalization function that prevents excessive model complexity–which may lead to overfitting–by smoothing the learned weights. We assigned class labels according to depression status and trained the model to predict depression in the dementia caregiver and general population datasets. We applied hyperparameters as default settings while changing a few of them (n_estimators = 3,000, scale_pos_weight = 1.5, nthread = 4, eta = 0.3, gamma = 0).

The LR algorithm classifies variables among binary classes based on a probability value ranging from 0 to 1 (14). This probability is compared to a threshold value, with classes being assigned to variables according to the result. In this study, we used the default threshold of 0.5 for classification. The L2 penalty was assigned in this model, and the solver was assigned as newton-cg.

The SVC algorithm is a clustering method based on the decision boundary, a standard used to cluster datasets into different groups. By setting the support vector, a training data point located closest to the decision boundary, the model attempts to maximize the distance between the support vector and the decision boundary (15). In the case of non-linear datasets, the boundary is set by means of a kernel, which maps the variables to a higher-dimensional feature space, allowing the model to learn the appropriate features. Thus, a kernel enables the clustering of more complex datasets that are not linearly separable. In the present study, the radial basis function (rbf) kernel was employed for mapping, and gamma was set to auto.

To identify the determinants of depression in dementia caregivers, we divided the final dataset into dementia caregiver and general population group each according to the “dementia caregiver's status of residence with the patient.” The size of the dataset of each group then resulted as (7,854, 33) as the standard variable (i.e., “dementia caregiver's status of residence with the patient”) was excluded. Then, we divided each dataset into a 9:1 ratio, 7,086:786. We put the former to train dataset and the latter to test dataset for all three machine learning algorithms. Among 33 variables, “depression experience” was set to the dependent variable, with the remaining 32 variables designated as independent. A 10-fold cross validation (CV) method was applied to each model in the training and test process to prevent overfitting. To ensure easy replicability, all other settings were as default. Upon completion of analyses, we selected the best model based on performance. The evaluation metrics used for model selection were accuracy (4), precision (5), recall (6), and F1-score (7). All metrics were calculated from the confusion matrix, which comprises true positive (TP), true negative (TN), false positive (FP), and false negative (FN) values. The four evaluation metric were calculated using the following formulas:


(4) $$\begin{array}{l} Accuracy=\frac{TP+TN}{TP+TN+FP+FN} \end{array}$$

(5) $$\begin{array}{l} Precision=\frac{TP}{TP+FP} \end{array}$$

(6) $$\begin{array}{l} Recall=\frac{TP}{TP+FN} \end{array}$$

(7) $$\begin{array}{l} F1-score=2\times \frac{\left(Precision\times Recall\right)}{\left(Precision\;+\;Recall\right)} \end{array}$$

### 2.5. Statistical analysis

To validate the accuracy of the results obtained from machine learning algorithms, we performed a hierarchical multivariate regression analysis to examine the effects of certain variables with the change of a statistically significant amount (e.g., R^2^ score). We validated the obtained ranks of depression causes by ordering the variables according to predicted feature importance. All variables were arranged into blocks of 10 according to significance, and the two least-significant variables were disregarded. The analysis comprised two phases: an in-group analysis, and a between-group analysis. In the former, we validated the ranking within each group by ordering the variables. In the latter, we verified the accuracy of the overall ranking by examining combinations between the groups. Based on the results from a hierarchical multivariate regression analysis, we performed a Welch's *t-*test on the high impact variables of dementia caregiver and general population each to verify the result of previous analysis.

### 2.6. Tools

The entire code for data preprocessing and machine learning algorithms was written in Python (version 3.9.7; scikit-learn, version 0.24.2). The entire code for statistical analyses was written in R (version 4.2.1).

## 3. Results

### 3.1. Machine learning classification performance

The performance of all classification algorithms is summarized in Tables 3, 4. For both dementia caregiver and general population datasets, XGBoost exhibited optimal performance. Although the model obtained lower recall and F1-score values than the other models in both cases, it achieved the best results in accuracy and precision. Because accuracy represents overall classification, whereas all other metrics only account for TPs, we assigned a higher weight to accuracy (e.g., Group 1+Group 2, Group 1+Group 2+Group 3).

**Table 3:** Classification performance on dementia caregiver dataset.

**Model**	**XGBoost**	**LR**	**SVC**
Accuracy	0.853	0.766	0.802
Precision	0.649	0.640	0.630
Recall	0.591	0.755	0.684
F1-score	0.608	0.653	0.646

**Table 4:** Classification performance on general population dataset.

**Model**	**XGBoost**	**LR**	**SVC**
Accuracy	0.915	0.797	0.855
Precision	0.655	0.603	0.592
Recall	0.580	0.763	0.651
F1-score	0.601	0.616	0.609

### 3.2. Feature importance of ML classifiers

**Figure 2:**
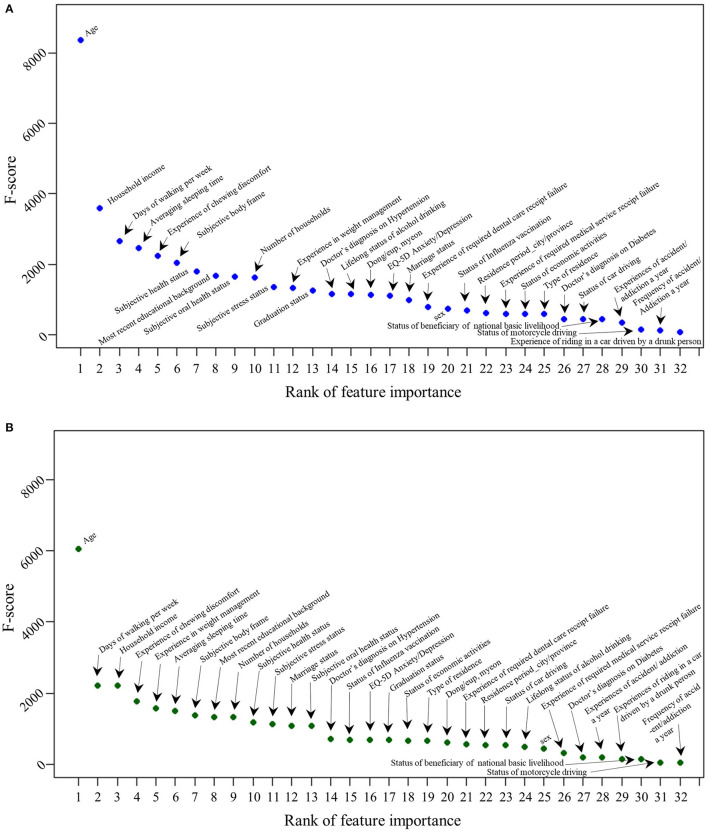
Feature importance of depression factors in dementia caregivers and general population (XGBoost). **(A)** Dementia caregiver. **(B)** General population.

The feature importance results obtained by the XGBoost model are graphed in Figure 2. Overall, the rankings appear to be largely similar among the two datasets. However, “sex” and “lifelong status of alcohol drink” are ranked higher within the dementia caregiver dataset, whereas “experience of weight management,” “status of economic activities,” and “type of residence” are ranked higher in the general population dataset.

### 3.3. Validation of feature importance by statistical model analysis

We conducted a hierarchical multivariate regression analysis to validate the results obtained in the previous section. First, we performed an in-group analysis to validate rankings within groups, as shown in Supplementary Tables 1, 2. For each group, the top five determinants were placed in the first block, the following two determinants were added to the second block, and the bottom three determinants were added to the third block. As new variables were introduced, the R^2^ value increased along with the size of determinants. Thus, we verified the accuracy of ranking within each group.

Subsequently, we performed a between-group analysis to validate the rankings of determinants between groups. Results are listed in Supplementary Tables 3, 4. We considered the five determinants that exhibited variability between the datasets: sex, lifelong status of alcohol drink, experience of weight management, status of economic activities, and type of residence. Within each block, all variables were placed according to rank of feature importance. To prevent the overpowering effect of high-impact variables, our analysis encompassed three iterations, with the number of Group 1 variables changing from the top 5 to the top 10.

Overall, the inter-group ranking was confirmed by a reduction in the increase of R^2^ scores with the addition of new variables. However, two exceptions exhibited a sizable increase in the R^2^ scores: “sex” and “status of economic activities.” Likewise, the combination of the two variables produced the highest R^2^ values. To further analyze the ranks of these variables in both datasets, we compared the change in R^2^ values between the two. In the dementia caregiver dataset, the change in R^2^ between “Group1 variables” and “Group1 variables + status of economic activities” was 0.007, and the change between “Group1 variables + status of economic activities” and “Group1 variables + status of economic activities + sex” was 0.0037. In the general population dataset, the change in R^2^ value between “Group1” variables and “Group1 variables + sex” was 0.0061, and the change between “Group1 variables + sex” and “Group1 variables + sex + status of economic activities” was 0.0053. From these results, we conclude that “sex” has a higher impact on depression than “status of economic activities” in dementia caregivers, whereas the reverse is true for the general population.

### 3.4. Statistical test

Because we found “sex” and “status of economic activities” to have a high impact on depression in dementia caregivers and the general population, respectively, we further verified the results with Welch's *t*-test, as shown in Supplementary Tables 5, 6. This test was performed to evaluate the difference in depression experiences in each group with respect to variables. As positive and negative responses regarding depression experiences were coded as 1 and 2, respectively, our objective was to determine which group yields a lower sample estimate. The results exhibited a difference among the datasets. In dementia caregiver group, women were found to experience depression more than men (*p* < 0.01). In general population group, a lack of economic activities was associated with depression (*p* < 0.01). Thus, the results obtained by XGBoost and the statistical model analysis were verified to be accurate.

## 4. Discussion

This study was conducted to identify determinants of the psychological burden experienced by dementia caregivers in South Korea. Results were obtained by machine learning algorithms and subsequently verified through a statistical model analysis. By allocating the data provided by the KDCA among dementia caregivers and general population groups, we were able to rank the determinants of depression within each group. Studies regarding the predictors of caregiver burden have been conducted for various illnesses. D'Amelio et al. investigated such predictors in Parkinson's Disease caregivers (16), and Coen et al. discovered comparable factors for Alzheimer's Disease (17). Similar to our study, Kim et al. examined the predictors of caregiver burden for dementia (18). Due to its advantages, many studies have employed machine learning to discover the predictors of psychological burdens. Prout et al. conducted a study to determine the predictors of psychological distress worldwide during the COVID-19 pandemic using machine learning algorithms (19). Likewise, Zhang et al. applied machine learning to evaluate the impact of predictors of postpartum depression in pregnant women (20).

After performing the classification task with three machine learning algorithms (i.e., “SVC,” “LR,” and “XGBoost”), we selected the XGBoost classifier, which exhibited the best performance. The XGBoost model obtained the feature importance of determinants of depression within the dementia caregiver and general population groups. Although the two groups exhibited similar overall rankings, they showed differences in the low-ranked variables (Figure 2).

To validate the results of the XGBoost classifier, we hierarchically allocated the determinants among three groups (“Group 1,” “Group 2,” “Group 3”), with 10 variables in each. Group 1, which comprised the 10 most significant determinants, had nine common variables, and the result dovetailed with prior findings on depression. First, “age” was determined to be the highest-ranked variable in both groups. Likewise, a prior study has also discovered a higher prevalence of depression among the elderly (21, 22). Particularly, the result aligns with the previous study that showed the high average age of dementia caregivers, which ranges from 43.6 to 71.8 (23). The age distribution of dementia caregivers accouns for the prominent impact of age on depression experience in the dementia caregiver group. “Days of walking per week” was also found to have a high impact on depression in both groups. It aligns with the prior finding on a positive correlation between physical activity and depression (24, 25), and the clinical effectiveness of walking as a treatment for depression (26). “Household income” and “most recent educational background” were also found to have a high impact on depression in both groups, aligning with prior findings on the positive relationship between socioeconomic status and depression (27). “Number of households” was another determinant that exhibited a high rank in both groups. Likewise, Sempungu et al. found that an increase in household size is associated with a lower prevalence of depression among the Korean population (28). Another high-ranking factor in both groups was the “experience of chewing discomfort.” A negative relationship between oral health and depression has been established in prior studies, where tooth loss was found to have a significant effect (29, 30). Although “average sleeping time” exhibited a high rank in both groups, the rank was slightly higher for dementia caregivers. Al-Abri found a strong bidirectional relationship between sleep deprivation and depression in the general population (31). Furthermore, Gao et al. discovered that dementia caregivers generally sleep 2.42–3.5 fewer hours per week with poorer sleep quality, than the general population (32), supporting a higher rank in dementia caregivers. “Subjective body frame” was another high-impact variable in both groups. Likewise, Richard et al. discovered a positive relationship between depression and body weight dissatisfaction in general populations (33). Finally, “subjective health status” appeared in Group 1 for both groups. Chang-Quan et al. found a similarly strong association between poor self-rated health status and depression among the elderly (34). However, “subjective oral health status” ranked 9^th^ in the dementia caregiver group, and 13^th^ in the general population group. Furthermore, “experience of weight management” ranked 5^th^ in general populations and 12^th^ in dementia caregivers. A strong correlation between depression and both variables was proven in previous studies (30, 35), however, a gap of ranks between dementia caregivers and general populations in them have been discovered in our study.

For Groups 2 and 3, a comparison between the two datasets produced divergent results. There were a few variables that showed a significant gap of ranks between the dementia caregiver and the general population group unlike Group 1. For example, “sex” ranked 20^th^ in the dementia caregiver group, and 25^th^ in the general population group. Moreover, “status of economic activities” ranked 24^th^ in dementia caregivers, and 18^th^ in general populations. In addition, “lifelong status of alcohol drinking” ranked 15^th^ in dementia caregivers and 24^th^ in general populations. Contrarily, some of the variables did not reflect a large gap between the two groups. For instance, “EQ-5D Anxiety/Depression” ranked 17^th^ in the dementia caregiver group, and 16^th^ in the general population group. Also, “Experience of required dental care receipt failure” ranked 19^th^ in the dementia caregiver group, and 21^st^ in the general population group. The rank of “Residence Period_City/Province” was the same as 22^nd^ in both population groups.

Furthermore, we performed a statistical model analysis to verify the accuracy of the aforementioned ranking results. In the in-group analysis, we assessed the rankings of determinants within each group. We constructed three blocks containing five, two, and three variables ordered by rank, respectively, and determined the pseudo R^2^ scores of all blocks, as listed in Supplementary Tables 1, 2. We verified the increase in scores with the addition of new variables in each block of both groups, thereby verifying the accuracy of the ranks obtained by XGBoost. We also found that the increase in R^2^ scores was higher in Block 2 than in Block 3 for both groups. Thus, we verified the hierarchy of variables between the two groups.

In the between-group analysis, we evaluated the ranks of determinants between the groups. By selecting the variables showing differences, we placed Group 1 variables in the first block, Group 2 variables in the second, and Group 3 variables in the third. Subsequently, we obtained the pseudo R^2^ scores of all blocks, as shown in Supplementary Tables 3, 4. Overall, we found the combination of all ten variables in Group 1 to produce the highest scores, demonstrating the high impact of the variables. Furthermore, the R^2^ score of Block 2 containing “sex” was highest, and “status of economic activities” was highest in Block 3 in dementia caregivers. These results were reversed in the general population group, wherein “status of economic activities” yielded the highest R^2^ score for Block 2, and “sex” produced the highest R^2^ score for Block 3.

Subsequently, we performed an additional statistical test on high-impact variables within each group, as shown in Supplementary Tables 5, 6. By performing a *t-*test, we found that women tend to experience depression more frequently in dementia caregivers, whereas the lack of economic activities was strongly associated with depression in general populations. As a result, we concluded that “sex” is the strongest determinant of depression in dementia caregivers, and “status of economic activities” is the strongest determinant of depression in the general population, considering the hierarchy of determinants resulted from XGBoost classifier (i.e., feature importance) and the result from statistical analyses (i.e., a hierarchical multivariate regression analysis, Welch's *t*-test) which have proven the rank of determinants once more.

The findings on the high Impact of determinants on depression In each group also dovetails with the previous studies. Previous study has discovered that the females suffer more than males from the caregiver burden of dementia patients (23, 36). A higher rank of “sex” in the caregivers can be interpreted in terms of it. Moreover, it has been found that the primary gender ratio in dementia caregiver participation is female (23). A predominant occupation of females in caregiver accounts for a high impact on depression experience. A high impact of “status of economic activities” on depression of the general population has also been proven. A positive association between depression and employment status in the general population has been indicated by previous studies (37, 38). Not just the risk of depression experience, but also the pattern of depression may vary depending on the employment status (38). The correlation aligns with our findings on the principal impact of “status of economic activities” on depression experience in the general population.

The present study was conducted to identify the determinants of depression among dementia caregivers. We used a large-scale dataset encompassing nationwide information from the general population of South Korea. We first employed three machine learning algorithms and subsequently validated the results obtained by the best-performing model. However, this study had several limitations. Because the survey respondents within the dataset represent the general population, a reduction in dataset size was inevitable when creating the dementia caregiver set. To supplement the reduction, we applied 10-fold CV in the machine learning process to eliminate bias and fully represent the dataset's characteristics prior to analysis. Furthermore, few variables were found to exhibit significant differences between the dementia caregiver and general population groups. The similarity among most determinants of depression can be attributed to the dataset, which has been collected from the general population. An additional study with an appropriate dataset may therefore be required to elucidate the differences in the causes of depression between the two groups.

Our study reveals an apparent difference in the main determinants of depression between the dementia caregiver and the general population, which indicates a necessity to conduct further research pertaining to dementia caregivers. The findings obtained by this study may serve as a basis for policy establishment to improve the mental health of dementia caregivers.

## Data availability statement

Publicly available datasets were analyzed in this study. This data can be found at: https://chs.kdca.go.kr/chs/ from Korea Centers for Disease Control and Prevention Data (KDCA).

## Author contributions

KC and JC designed the model and the computational framework, analyzed the data, and wrote the manuscript with input from all authors. KC, JC, and SH carried out the implementation. KC performed the calculations. SH conceived the study and was in charge of overall direction and planning. All authors contributed to the article and approved the submitted version.
