# Supplementary material for: Validation of depression determinants in caregivers of dementia patients with machine learning algorithms and statistical model
Source: Front Med (Lausanne). 2023 Feb 2;10:1095385. doi: 10.3389/fmed.2023.1095385 (PMC9932916; doi:10.3389/fmed.2023.1095385)
Supplement: Supplementary file 1 [file Data_Sheet_1.PDF]

Supplementary Material

Validation of depression determinants in caregivers of dementia patients with machine learning algorithms and statistical model

Kangrim Cho, Junggu Choi, Sanghoon Han*

*** Correspondence: Sanghoon Han**: sanghoon.han@yonsei.ac.kr

Table 1. Results of in-group statistical model analysis in dementia caregiver dataset

Groups involved	Block	Variable	Pseudo R^2
Group1	1st block	Age, Household Income, Days of Walking per week, Average Sleeping Time, Experience of Chewing Discomfort	0.0309
	2nd block	Age, Household Income, Days of Walking per week, Average Sleeping Time, Experience of Chewing Discomfort, Subjective Body Frame, Subjective Health Status	0.0722
	3rd block	Age, Household Income, Days of Walking per week, Average Sleeping Time, Experience of Chewing Discomfort, Subjective Body Frame, Subjective Health Status, Most Recent Educational Background, Subjective Oral Health Status, Number of Households	0.0734
Group2	1st block	Subjective Stress Status, Experience of Weight Management, Graduation Status, Doctor's Diagnosis of Hypertension, Lifelong Status of Alcohol Drinking	0.1400
	2nd block	Subjective Stress Status, Experience of Weight Management, Graduation Status, Doctor's Diagnosis of Hypertension, Lifelong Status of Alcohol Drinking, dong/eup, myeon, EQ-5D Anxiety/Depression	0.2284
	3rd block	Subjective Stress Status, Experience of Weight Management, Graduation Status, Doctor's Diagnosis of Hypertension, Lifelong Status of Alcohol Drinking, dong/eup, myeon, EQ-5D Anxiety/Depression, Marriage Status, Experience of Required Dental Care Receipt Failure, Sex	0.2347
Group3	1st block	Status of Influenza Vaccination, Residence Period_City/Province, Experience of Required Medical Service Receipt Failure, Status of Economic Activities, Type of Residence	0.0499
	2nd block	Status of Influenza Vaccination, Residence Period_City/Province, Experience of Required Medical Service Receipt Failure, Status of Economic Activities, Type of Residence, Doctor's Diagnosis on Diabetes, Status of Car Driving	0.0530
	3rd block	Status of Influenza Vaccination, Residence Period_City/Province, Experience of Required Medical Service Receipt Failure, Status of Economic Activities, Type of Residence, Doctor's Diagnosis on Diabetes, Status of Car Driving, Status of Beneficiary of National Basic Livelihood, Experiences of Accident/Addiction a Year, Status of Motorcycle Driving	0.0646

Table 2. Results of in-group statistical model analysis in general population dataset

Groups involved	Block	Variable	Pseudo R^2
Group1	1st block	Age, Days of Walking per week, Household Income, Experience of Chewing Discomfort, Experience of Weight Management	0.0319
	2nd block	Age, Days of Walking per week, Household Income, Experience of Chewing Discomfort, Experience of Weight Management, Average Sleeping Time, Subjective Body Frame	0.0334
	3rd block	Age, Days of Walking per week, Household Income, Experience of Chewing Discomfort, Experience of Weight Management, Average Sleeping Time, Subjective Body Frame, Most Recent Educational Background, Number of Households, Subjective Health Status	0.0762
Group2	1st block	Subjective Stress Status, Marriage Status, Subjective Oral Health Status, Doctor's Diagnosis of Hypertension, Status of Influenza Vaccination	0.0606
	2nd block	Subjective Stress Status, Marriage Status, Subjective Oral Health Status, Doctor's Diagnosis of Hypertension, Status of Influenza Vaccination, EQ-5D Anxiety/Depression, Graduation Status	0.1770
	3rd block	Subjective Stress Status, Marriage Status, Subjective Oral Health Status, Doctor's Diagnosis of Hypertension, Status of Influenza Vaccination, EQ-5D Anxiety/Depression, Graduation Status, Status of Economic Activities, Type of Residence, dong/eup, myeon	0.2316
Group3	1st block	Experience of Required Dental Care Receipt Failure, sex, Status of Car Driving, Lifelong Status of Alcohol Drinking, Residence Period_City/Province	0.0383
	2nd block	Experience of Required Dental Care Receipt Failure, sex, Status of Car Driving, Lifelong Status of Alcohol Drinking, Residence Period_City/Province, Experience of Required Medical Service Receipt Failure, Doctor's Diagnosis on Diabetes	0.0526
	3rd block	Experience of Required Dental Care Receipt Failure, sex, Status of Car Driving, Lifelong Status of Alcohol Drinking, Residence Period_City/Province, Experience of Required Medical Service Receipt Failure, Doctor's Diagnosis on Diabetes, Experiences of Accident/Addiction a Year, Experience of Riding in a Car Driven by a Drunk Person, Status of Beneficiary of National Basic Livelihood	0.0664

Table 3. Results of between-group statistical model analysis in dementia caregiver dataset

Groups involved	Block	Variable	Pseudo R^2	Groups involved	Block	Variable	Pseudo R^2
Group1	1st block	Age, Household Income, Days of Walking per week, Average Sleeping Time, Experience of Chewing Discomfort, Subjective Body Frame, Subjective Health Status, Most Recent Educational Background, Subjective Oral Health Status, Number of Households	0.0734	Group1	1st block	Age, Household Income, Days of Walking per week, Average Sleeping Time, Experience of Chewing Discomfort, Subjective Body Frame, Subjective Health Status	0.0722
Group2	2nd block	Experience in Weight Management	0.0762	Group2	2nd block	Sex	0.0782
Group3	3rd block	Type of Residence	0.0764	Group3	3rd block	Status of Economic Activities	0.0835
Group1	1st block	Age, Household Income, Days of Walking per week, Average Sleeping Time, Experience of Chewing Discomfort, Subjective Body Frame, Subjective Health Status, Most Recent Educational Background, Subjective Oral Health Status, Number of Households	0.0734	Group1	1st block	Age, Household Income, Days of Walking per week, Average Sleeping Time, Experience of Chewing Discomfort, Subjective Body Frame, Subjective Health Status	0.0722
Group2	2nd block	Experience in Weight Management	0.0762	Group2	2nd block	Lifelong Status of Alcohol Drinking	0.0723
Group3	3rd block	Status of Economic Activities	0.0832	Group3	3rd block	Type of Residence	0.0725
Group1	1st block	Age, Household Income, Days of Walking per week, Average Sleeping Time, Experience of Chewing Discomfort, Subjective Body Frame, Subjective Health Status, Most Recent Educational Background, Subjective Oral Health Status, Number of Households	0.0734	Group1	1st block	Age, Household Income, Days of Walking per week, Average Sleeping Time, Experience of Chewing Discomfort, Subjective Body Frame, Subjective Health Status	0.0722
Group2	2nd block	Sex	0.0795	Group2	2nd block	Lifelong Status of Alcohol Drinking	0.0723
Group3	3rd block	Type of Residence	0.0795	Group3	3rd block	Status of Economic Activities	0.0798
Group1	1st block	Age, Household Income, Days of Walking per week, Average Sleeping Time, Experience of Chewing Discomfort, Subjective Body Frame, Subjective Health Status, Most Recent Educational Background, Subjective Oral Health Status, Number of Households	0.0734	Group1	1st block	Age, Household Income, Days of Walking per week, Average Sleeping Time, Experience of Chewing Discomfort	0.0309
Group2	2nd block	Sex	0.0795	Group2	2nd block	Experience in Weight Management	0.0332
Group3	3rd block	Status of Economic Activities	0.0848	Group3	3rd block	Type of Residence	0.0332
Group1	1st block	Age, Household Income, Days of Walking per week, Average Sleeping Time, Experience of Chewing Discomfort, Subjective Body Frame, Subjective Health Status, Most Recent Educational Background, Subjective Oral Health Status, Number of Households	0.0734	Group1	1st block	Age, Household Income, Days of Walking per week, Average Sleeping Time, Experience of Chewing Discomfort	0.0309
Group2	2nd block	Lifelong Status of Alcohol Drinking	0.0735	Group2	2nd block	Experience in Weight Management	0.0332

Group3	3rd block	Type of Residence	0.0737	Group3	3rd block	Status of Economic Activities	0.0487
Group1	1st block	Age, Household Income, Days of Walking per week, Average Sleeping Time, Experience of Chewing Discomfort, Subjective Body Frame, Subjective Health Status, Most Recent Educational Background, Subjective Oral Health Status, Number of Households	0.0734	Group1	1st block	Age, Household Income, Days of Walking per week, Average Sleeping Time, Experience of Chewing Discomfort	0.0309
Group2	2nd block	Lifelong Status of Alcohol Drinking	0.0735	Group2	2nd block	Sex	0.0782
Group3	3rd block	Status of Economic Activities	0.0808	Group3	3rd block	Type of Residence	0.0394
Group1	1st block	Age, Household Income, Days of Walking per week, Average Sleeping Time, Experience of Chewing Discomfort, Subjective Body Frame, Subjective Health Status	0.0722	Group1	1st block	Age, Household Income, Days of Walking per week, Average Sleeping Time, Experience of Chewing Discomfort	0.0309
Group2	2nd block	Experience in Weight Management	0.0750	Group2	2nd block	Sex	0.0782
Group3	3rd block	Type of Residence	0.0750	Group3	3rd block	Status of Economic Activities	0.0512
Group1	1st block	Age, Household Income, Days of Walking per week, Average Sleeping Time, Experience of Chewing Discomfort, Subjective Body Frame, Subjective Health Status	0.0722	Group1	1st block	Age, Household Income, Days of Walking per week, Average Sleeping Time, Experience of Chewing Discomfort	0.0309
Group2	2nd block	Experience in Weight Management	0.0750	Group2	2nd block	Lifelong Status of Alcohol Drinking	0.0309
Group3	3rd block	Status of Economic Activities	0.0821	Group3	3rd block	Type of Residence	0.0310
Group1	1st block	Age, Household Income, Days of Walking per week, Average Sleeping Time, Experience of Chewing Discomfort, Subjective Body Frame, Subjective Health Status	0.0722	Group1	1st block	Age, Household Income, Days of Walking per week, Average Sleeping Time, Experience of Chewing Discomfort	0.0309
Group2	2nd block	Sex	0.0782	Group2	2nd block	Lifelong Status of Alcohol Drinking	0.0309
Group3	3rd block	Type of Residence	0.0783	Group3	3rd block	Status of Economic Activities	0.0467

Table 4. Results of between-group statistical model analysis in general population dataset

Groups involved	Block	Variable	Pseudo R^2	Groups involved	Block	Variable	Pseudo R^2
Group1	1st block	Age, Days of Walking per week, Household Income, Experience of Chewing Discomfort, Experience of Weight Management, Average Sleeping Time, Subjective Body Frame, Most Recent Educational Background, Number of Households, Subjective Health Status	0.0762	Group1	1st block	Age, Days of Walking per week, Household Income, Experience of Chewing Discomfort, Experience of Weight Management, Average Sleeping Time, Subjective Body Frame	0.0334
Group2	2nd block	Subjective Oral Health Status	0.0762	Group2	2nd block	Type of Residence	0.0335
Group3	3rd block	Sex	0.0816	Group3	3rd block	Lifelong Status of Alcohol Drinking	0.0335
Group1	1st block	Age, Days of Walking per week, Household Income, Experience of Chewing Discomfort, Experience of Weight Management, Average Sleeping Time, Subjective Body Frame, Most Recent Educational Background, Number of Households, Subjective Health Status	0.0762	Group1	1st block	Age, Days of Walking per week, Household Income, Experience of Chewing Discomfort, Experience of Weight Management, Average Sleeping Time, Subjective Body Frame	0.0334
Group2	2nd block	Subjective Oral Health Status	0.0762	Group2	2nd block	Status of Economic Activities	0.0489
Group3	3rd block	Lifelong Status of Alcohol Drinking	0.0763	Group3	3rd block	Sex	0.0531
Group1	1st block	Age, Days of Walking per week, Household Income, Experience of Chewing Discomfort, Experience of Weight Management, Average Sleeping Time, Subjective Body Frame, Most Recent Educational Background, Number of Households, Subjective Health Status	0.0762	Group1	1st block	Age, Days of Walking per week, Household Income, Experience of Chewing Discomfort, Experience of Weight Management, Average Sleeping Time, Subjective Body Frame	0.0334
Group2	2nd block	Type of Residence	0.0764	Group2	2nd block	Status of Economic Activities	0.0489
Group3	3rd block	Sex	0.0816	Group3	3rd block	Lifelong Status of Alcohol Drinking	0.0490
Group1	1st block	Age, Days of Walking per week, Household Income, Experience of Chewing Discomfort, Experience of Weight Management, Average Sleeping Time, Subjective Body Frame, Most Recent Educational Background, Number of Households, Subjective Health Status	0.0762	Group1	1st block	Age, Days of Walking per week, Household Income, Experience of Chewing Discomfort, Experience of Weight Management	0.0319
Group2	2nd block	Type of Residence	0.0764	Group2	2nd block	Subjective Oral Health Status	0.0330
Group3	3rd block	Lifelong Status of Alcohol Drinking	0.0765	Group3	3rd block	Sex	0.0411
Group1	1st block	Age, Days of Walking per week, Household Income, Experience of Chewing Discomfort, Experience of Weight Management, Average Sleeping Time, Subjective Body Frame, Most Recent Educational Background, Number of Households, Subjective Health Status	0.0762	Group1	1st block	Age, Days of Walking per week, Household Income, Experience of Chewing Discomfort, Experience of Weight Management	0.0319
Group2	2nd block	Status of Economic Activities	0.0832	Group2	2nd block	Subjective Oral Health Status	0.0330

Group3	3rd block	Sex	0.0869	Group3	3rd block	Lifelong Status of Alcohol Drinking	0.0331
Group1	1st block	Age, Days of Walking per week, Household Income, Experience of Chewing Discomfort, Experience of Weight Management, Average Sleeping Time, Subjective Body Frame, Most Recent Educational Background, Number of Households, Subjective Health Status	0.0762	Group1	1st block	Age, Days of Walking per week, Household Income, Experience of Chewing Discomfort, Experience of Weight Management	0.0319
Group2	2nd block	Status of Economic Activities	0.0832	Group2	2nd block	Type of Residence	0.0319
Group3	3rd block	Lifelong Status of Alcohol Drinking	0.0835	Group3	3rd block	Sex	0.0398
Group1	1st block	Age, Days of Walking per week, Household Income, Experience of Chewing Discomfort, Experience of Weight Management, Average Sleeping Time, Subjective Body Frame	0.0334	Group1	1st block	Age, Days of Walking per week, Household Income, Experience of Chewing Discomfort, Experience of Weight Management	0.0319
Group2	2nd block	Subjective Oral Health Status	0.0346	Group2	2nd block	Type of Residence	0.0319
Group3	3rd block	Sex	0.0426	Group3	3rd block	Lifelong Status of Alcohol Drinking	0.0320
Group1	1st block	Age, Days of Walking per week, Household Income, Experience of Chewing Discomfort, Experience of Weight Management, Average Sleeping Time, Subjective Body Frame	0.0334	Group1	1st block	Age, Days of Walking per week, Household Income, Experience of Chewing Discomfort, Experience of Weight Management	0.0319
Group2	2nd block	Subjective Oral Health Status	0.0346	Group2	2nd block	Status of Economic Activities	0.0470
Group3	3rd block	Lifelong Status of Alcohol Drinking	0.0346	Group3	3rd block	Sex	0.0513
Group1	1st block	Age, Days of Walking per week, Household Income, Experience of Chewing Discomfort, Experience of Weight Management, Average Sleeping Time, Subjective Body Frame	0.0334	Group1	1st block	Age, Days of Walking per week, Household Income, Experience of Chewing Discomfort, Experience of Weight Management	0.0319
Group2	2nd block	Type of Residence	0.0335	Group2	2nd block	Status of Economic Activities	0.0470
Group3	3rd block	Sex	0.0413	Group3	3rd block	Lifelong Status of Alcohol Drinking	0.0470

Table 5. Depression experience by gender in dementia caregiver dataset

Classification	n	Sample Estimates	95% CI	P-value	t(df)
Men	3071	1.899381	0.0371 to 0.0665	5.986-e12	6.891(7325.6)
Women	4783	1.847585			

Table 6. Depression experience by economic activity in general population dataset

Classification	n	Sample Estimates	95% CI	P-value	t(df)
Having Economic Activities	4157	1.949964	0.0428 to 0.0665	< 2.2-e16	9.008(6589.7)
Not Having Economic Activities	3697	1.895321			
